# Co-administration of ursodeoxycholic acid with rosuvastatin/ezetimibe in a non-alcoholic fatty liver disease model

**DOI:** 10.1093/gastro/goac037

**Published:** 2022-08-13

**Authors:** Sang Hyun Seo, Da Hyun Lee, Yu Seol Lee, Kyung Joo Cho, Hye Jung Park, Hye Won Lee, Beom Kyung Kim, Jun Yong Park, Do Young Kim, Sang Hoon Ahn, Soo Han Bae, Seung Up Kim

**Affiliations:** Department of Internal Medicine, Graduate School of Medicine Science, Brain Korea 21 Project, Yonsei University College of Medicine, Seoul, Republic of Korea; Yonsei Liver Center, Severance Hospital, Seoul, Republic of Korea; Severance Biomedical Science Institute, Graduate School of Medical Science, Brain Korea 21 Project, Yonsei University College of Medicine, Seoul, Republic of Korea; Severance Biomedical Science Institute, Yonsei Biomedical Research Institute, Yonsei University College of Medicine, Seoul, Republic of Korea; Severance Biomedical Science Institute, Graduate School of Medical Science, Brain Korea 21 Project, Yonsei University College of Medicine, Seoul, Republic of Korea; Severance Biomedical Science Institute, Yonsei Biomedical Research Institute, Yonsei University College of Medicine, Seoul, Republic of Korea; Department of Internal Medicine, Graduate School of Medicine Science, Brain Korea 21 Project, Yonsei University College of Medicine, Seoul, Republic of Korea; Yonsei Liver Center, Severance Hospital, Seoul, Republic of Korea; Yonsei Liver Center, Severance Hospital, Seoul, Republic of Korea; Yonsei Liver Center, Severance Hospital, Seoul, Republic of Korea; Department of Internal Medicine, Institute of Gastroenterology, Yonsei University College of Medicine, Seoul, Republic of Korea; Yonsei Liver Center, Severance Hospital, Seoul, Republic of Korea; Department of Internal Medicine, Institute of Gastroenterology, Yonsei University College of Medicine, Seoul, Republic of Korea; Yonsei Liver Center, Severance Hospital, Seoul, Republic of Korea; Department of Internal Medicine, Institute of Gastroenterology, Yonsei University College of Medicine, Seoul, Republic of Korea; Yonsei Liver Center, Severance Hospital, Seoul, Republic of Korea; Department of Internal Medicine, Institute of Gastroenterology, Yonsei University College of Medicine, Seoul, Republic of Korea; Yonsei Liver Center, Severance Hospital, Seoul, Republic of Korea; Department of Internal Medicine, Institute of Gastroenterology, Yonsei University College of Medicine, Seoul, Republic of Korea; Severance Biomedical Science Institute, Graduate School of Medical Science, Brain Korea 21 Project, Yonsei University College of Medicine, Seoul, Republic of Korea; Severance Biomedical Science Institute, Yonsei Biomedical Research Institute, Yonsei University College of Medicine, Seoul, Republic of Korea; Yonsei Liver Center, Severance Hospital, Seoul, Republic of Korea; Department of Internal Medicine, Institute of Gastroenterology, Yonsei University College of Medicine, Seoul, Republic of Korea

**Keywords:** non-alcoholic fatty liver disease, non-alcoholic steatohepatitis, ursodeoxycholic acid, rosuvastatin, ezetimibe

## Abstract

**Background:**

Ursodeoxycholic acid (UDCA), statins, and ezetimibe (EZE) have demonstrated beneficial effects against non-alcoholic fatty liver disease (NAFLD). We investigated the efficacy of the combination of UDCA and the mix of rosuvastatin (RSV)/EZE in the treatment of NAFLD.

**Methods:**

NAFLD mouse models were developed by injecting thioacetamide, fasting, and high-carbohydrate refeeding, high-fat diet, and choline-deficient L-amino acid-defined high-fat diet (CDAHFD). Low-dose UDCA (L-UDCA; 15 mg/kg) or high-dose UDCA (H-UDCA; 30 mg/kg) was administered with RSV/EZE. We also employed an *in vitro* model of NAFLD developed using palmitic acid-treated Hepa1c1c7 cells.

**Results:**

Co-administration of RSV/EZE with UDCA significantly decreased the collagen accumulation, serum alanine aminotransferase (ALT) levels, and mRNA levels of fibrosis-related markers than those observed in the vehicle group in thioacetamide-treated mice (all *P* < 0.01). In addition, in the group fasted and refed with a high-carbohydrate diet, UDCA/RSV/EZE treatment decreased the number of apoptotic cells and serum ALT levels compared with those observed in the vehicle group (all *P* < 0.05). Subsequently, H-UDCA/RSV/EZE treatment decreased the number of ballooned hepatocytes and stearoyl-CoA desaturase 1 (*SCD-1*) mRNA levels (*P* = 0.027) in the liver of high-fat diet-fed mice compared with those observed in the vehicle group. In the CDAHFD-fed mouse model, UDCA/RSV/EZE significantly attenuated collagen accumulation and fibrosis-related markers compared to those observed in the vehicle group (all *P* < 0.05). In addition, UDCA/RSV/EZE treatment significantly restored cell survival and decreased the protein levels of apoptosis-related markers compared to RSV/EZE treatment in palmitic acid-treated Hepa1c1c7 cells (all *P* < 0.05).

**Conclusion:**

Combination therapy involving UDCA and RSV/EZE may be a novel strategy for potent inhibition of NAFLD progression.

## Introduction

Non-alcoholic fatty liver disease (NAFLD) is the most common cause of chronic liver disease worldwide. NAFLD encompasses the simple deposition of adipose tissue to more progressive steatosis with associated hepatitis, fibrosis, cirrhosis, and hepatocellular carcinoma [1]. A recent study showed that 34% of the adult population in the USA have excessive fat accumulation in the liver, mostly unrelated to alcohol intake [2]. In particular, the incidence of NAFLD-related hepatocellular carcinoma is increasing in the USA, with an increased number of cases expected by 2030 [3]. In addition, it is now well known that NAFLD is the most common liver disease in Korea, largely due to the considerable increase in metabolic disorders such as obesity and diabetes, comparable to the situation in the Western countries [4].

Currently, NAFLD is considered to be modified by a variety of components that act on susceptible genes and epigenetic backgrounds, but the specific mechanism remains unclear [5]. Currently, there is no government-approved drug for the treatment of NAFLD; therefore, it is necessary to identify the pathogenesis of NAFLD in the pursuit of new therapeutic targets [6]. In addition, although recent studies are moving rapidly toward combination therapies, there are also single drugs with multiple cellular or molecular targets of action that are under evaluation [7].

Ursodeoxycholic acid (UDCA) is an approved drug for the treatment of primary biliary cirrhosis, which is also used to treat several other cholestatic diseases [8]. UDCA is a bile acid formed in the liver and demonstrates antiapoptotic, cytoprotective, antioxidative and immunomodulatory properties [9, 10]. A recent study demonstrated that 2 years of UDCA treatment effectively reduced liver dysfunction in Indian patients with NAFLD [11] and a high dose of UDCA (H-UDCA) reduced alanine aminotransferase (ALT) levels in patients with non-alcoholic steatohepatitis (NASH), suggesting numerous hepatoprotective effects of the drug [12]. Moreover, UDCA prevents some deficiencies as signaling molecules, reduces the cholesterol content of bile, promotes the nuclear receptor in the liver, and protects the liver against retained bile acid in cholestatic diseases [13].

In addition, UDCA significantly regulates hydrophobic bile acids, such as lithocholic acid and deoxycholic acid conjugates, via reduction of cytotoxicity [14]. Moreover, UDCA significantly reduces systemic indole levels via hydroxylation and glucuronidation along with a decrease in the abundance of *Lactobacillus* and *Bifidobacterium* [15]. Hence, UDCA effectively improves liver function via bile acid regulation, metabolism regulation, and relevant microbiome remodeling, as well as by exerting antioxidant effects similar to those of vitamin E [16, 17]. However, the efficacy of UDCA is still controversial with respect to NASH [11, 18, 19]. Hence, it is necessary to demonstrate the potential mechanism of action of UDCA in NAFLD.

Combinative administration of rosuvastatin/ezetimibe (RSV/EZE) is an adjunctive therapy to diet control for the management of primary hypercholesterolemia among adults in numerous countries worldwide [20]. In previous studies, EZE reduced hepatic steatosis and dyslipidemia in a mouse model [21] and decreased the NAFLD activity score [22] and lipid accumulation [23]. In addition, various studies have demonstrated the significant low-density lipoprotein cholesterol lowering ability and cardiovascular event prevention effect of EZE–statin combination treatment [24, 25]. Therefore, it is anticipated that RSV/EZE treatment will have positive effects in patients with NAFLD.

Thus, the aim of this study was to evaluate the therapeutic effect of the combined use of H-UDCA or low-dose UDCA (L-UDCA) and a fixed dose of RSV/EZE. Therefore, we conducted a combination therapy of UDCA and RSV/EZE to evaluate its antilipogenic and antifibrotic effects on mouse models of NAFLD, including thioacetamide (TAA) injection, fasting and high-carbohydrate diet (HCD) refeeding, high-fat diet (HFD), and choline-deficient L-amino acid-defined high-fat diet (CDAHFD)-fed mouse models, and palmitic acid (PA)-induced lipotoxicity *in vitro* model of NAFLD.

## Material and methods

### Animal model

All experiments involving live mice were performed according to the Guidelines and Regulations for the Care and Use of Laboratory Animals in the Association for Assessment and Accreditation of Laboratory Animal Care, International (AAALAC)-accredited facilities, and were approved by the Animal Policy and Welfare Committee of the Yonsei University College of Medicine (Permit number: 2019–0149). We purchased 6-week-old C57BL/6J male mice from Japan SLC, Inc. (Hamamatsu, Japan). All mice had free access to water and food in rooms maintained at 23 ± 2°C with a 12-h light/12-h dark cycle and 50%–70% humidity. The mixture of phosphate buffered saline (PBS) and 8% dimethyl sulfoxide (DMSO), combination of RSV/EZE mix (total amount = 1 mg/kg; ratio = 1:2) with L-UDCA (15 mg/kg), and combination of RSV/EZE mix with H-UDCA (30 mg/kg) were oral administered in control (*n* = 5) and vehicle (*n* = 4) groups, L-UDCA/RSV/EZE group (*n* = 5), and H-UDCA/RSV/EZE group (*n* = 5). After 5 min, three non-control groups were treated with TAA (120 mg/kg) by intraperitoneal injection. All treatments were performed thrice per week for 3 weeks. Although we used five mice in each group, one of the vehicle subjects died during the experiment.

For fasting and refeeding with a HCD (#102235; Dyets, Bethlehem, Pennsylvania, USA) mouse model, C57BL6/J male mice were divided into a non-fasting group (control group, *n* = 4) and three groups refed with a HCD after 24 h of fasting (vehicle group, *n* = 4; L-UDCA/RSV/EZE group, *n* = 4; H-UDCA/RSV/EZE group, *n* = 5). In the control group, PBS/DMSO was administered 18 h prior to sacrifice. In three fasting groups, mice were administered with PBS/DMSO, L-UDCA/RSV/EZE, and H-UDCA/RSV/EZE 5 min prior to HCD refeeding. After 18 h of refeeding, mice in all groups were sacrificed. Five mice were used in each group, but four remained in the control, vector, and L-UDCA/RSV/EZE groups for statistical analysis excluding death during the experiment. C57BL/6J male mice were fed with a standard chow diet (#5053; LabDiet, St. Louis, Missouri, USA) or HFD, prepared by Research Diets, Inc. (#D12492; New Brunswick, New Jersey, USA) for 13 weeks. The mice were divided into four groups: chow-fed mice treated with PBS/DMSO (control group, *n* = 5), HFD-fed mice treated with PBS/DMSO (vehicle group, *n* = 5), and HFD-fed mice treated with L-UDCA/RSV/EZE or H-UDCA/RSV/EZE (each group, *n* = 5) thrice per week for 5 weeks before being sacrificed. Oral glucose tolerance test (OGTT) was performed during the last week of the experiment.

C57BL6/J mice were fed a standard chow diet or CDAHFD prepared by Research Diets, Inc. (#A06071302) for 8 weeks. After 4 weeks of chow or CDAHFD feeding, mice were divided into four groups (each group, *n* = 5): control group (chow-fed mice treated with PBS/DMSO), vehicle group (CDAHFD-fed mice treated with PBS/DMSO), L-UDCA/RSV/EZE group, and H-UDCA/RSV/EZE group (CDAHFD-fed mice treated with L-UDCA/RSV/EZE or H-UDCA/RSV/EZE). Mice in each group received treatment thrice per week for 4 weeks before sacrificed.

### Cell line

Hepa1c1c7 cells (22026; Korean Cell Line Bank, Seoul, Korea) were maintained in minimum essential medium (MEM; LM007-01; Welgene, Daegu, Korea) supplemented with 10% fetal bovine serum and 1% penicillin-streptomycin in a 5% CO_2_ atmosphere at 37°C.

### Serum biochemical assay

Mouse serum was harvested following the centrifugation of clotted blood samples and examined for biochemical parameters—ALT levels—using an automated clinical chemical analyser (#3250; Fujiflim, Tokyo, Japan).

### Histological analysis

The specimens embedded in paraffin blocks were sectioned into 5-μm slices. Then, they were processed by conventional hematoxylin and eosin (H&E) staining to evaluate general tissue morphology and Masson’s trichrome staining to evaluate fibrosis. The stained specimens were inspected under a microscope (Olympus, Tokyo, Japan). The oil red O working solution was composed of 0.5% oil red O solution in isopropanol and deionized water at a ratio of 3:2. For quantification, tissues were fixed with 4% formaldehyde at 25°C for 15 min. Thereafter, the tissues were stained with oil red O working solution for 1 h and washed with deionized water to remove any unbound dye. Then, 1 mL of isopropanol was added to the slides for 5 min. For the terminal deoxynucleotidyl transferase dUTP nick end-labeling (TUNEL) assay, a Click-iT Plus TUNEL assay kit (Promega Corporation, Madison, WI, USA) was used according to the manufacturer’s instructions. All sections were evaluated using a Zeiss LSM 700 confocal microscope (Carl Zeiss, Oberkochen, Germany).

### RNA isolation and real-time PCR amplification

Total Ribonucleic Acid (RNA) was isolated from tissues using TRIzol^®^ reagent (#TR 118; Molecular Research Center, Cincinnati, Ohio, USA). RNA (1 μg) was reverse-transcribed into cDNA using random hexamer primers and a cDNA synthesis kit (#RR036A; Takara, Kusatsu, Shiga, Japan). The resulting cDNA was subjected to quantitative Polymerase Chain Reaction (PCR) analysis using SYBR^®^ Green (#4367659; Applied Biosystems, Waltham, Massachusetts, USA) and mouse-specific primer pairs (forward and reverse). Primer sequences for mouse cDNAs are described in Table 1. The 18S ribosomal RNA was used as an internal control. The PCR reaction was initiated by heating at 55°C for 2 min, followed by heating at 95°C for 10 min. Then, 40 amplification cycles were performed with denaturation at 95°C for 15 s. Annealing and extension process is at 60°C for 1 min. The expression of specific genes was quantified using the 2^–ΔΔ^^*Ct*^ method.

**Table 1.** Primer sequences used in quantitative PCR

Gene	Forward sequence	Reverse sequence
SMA	CTGACAGAGGCACCACTGAA	CATCTCCAGAGTCCAGCACA
Col1a1	GAGCGGAGAGTACTGGATCG	GCTTCTTTTCCTTGGGGTTC
TGF-β	TTGCTTCAGCTCCACAGAGA	TGGTTGTAGAGGGCCAAGGAC
ACTA2	GACTACTGCCGAGCGTGA	GCTGTTAGGTGGTTTCGTGG
SREBP-1c	GGAGCCATGGATTGCACATT	GGCCCGGGAAGTCACTGT
FAS	GCTGCGGAAACTTCAGGAAAT	AGAGACGTCACTCCTGGACTT
SCD-1	AGAGACGTGTCACTCCTGGACTT	TAGCCTGTAAAAGATTTCTGCAAACC
18S	CGCTCCCAAGATCCAACTAC	CTGAGAAACGGCTACCACATC

### Western blot

For protein immunoblot analysis, mouse liver tissue lysates were subjected to sodium dodecyl sulfate polyacrylamide gel electrophoresis. The separated proteins were transferred to polyvinylidene difluoride membranes (L-IPVH 00010; Merck Millipore, Burlington, Massachusetts, USA). The membranes were incubated with antibodies against smooth muscle actin (SMA; ab5694; Abcam, Cambridge, England, UK), cleaved Poly (ADP-ribose) polymerase (c-PARP; 9544S; Cell Signaling Technology, Danvers, Massachusetts, USA), cleaved caspase 3 (c-Caspase 3; 9661S; Cell Signaling Technology), and β-actin (sc-47778; Santa Cruz Biotechnology, Dallas, Texas, USA) at 4°C overnight. Subsequently, the membranes were incubated with secondary antibodies at room temperature for 1 h. The blots were developed using an enhanced chemiluminescence solution (#34580; Thermo Fisher Scientific, Waltham, Massachusetts, USA).

### MTT assay for cell viability

Hepa1c1c7 cells were seeded at 2 × 10^3^ cells/well in a final volume of 100 μL in 96-well plates. After 24 h, the cells were treated with 500 µM bovine serum albumin (BSA) or PA (Sigma Aldrich, St. Louis, Missouri, USA) for 18 h. Cell viability was detected using a CellTiter-Glo luminescent cell viability assay kit (G7570; Promega Corporation) according to the manufacturer’s instructions.

### Saturated fatty acid treatment

PA was dissolved in isopropyl alcohol at a stock concentration of 160 mM. For the treatment of saturated fatty acid, the cells were incubated with 500 μM PA in Dulbecco's modified Eagle medium containing 1% BSA for 18 h to ensure a physiologically suitable ratio between the bound and unbound free fatty acids in the medium [26–29].

### Statistical analysis

All statistical analyses were performed using two-tailed Student’s *t*-tests for comparison between two groups or one-way analysis of variance with Tukey’s honest significant difference post-hoc analysis for multiple comparisons (SPSS 21.0 K for Windows; SPSS, Chicago, IL, USA). Statistical significance was set at *P* < 0.05. GraphPad Prism 9.0 (GraphPad, La Jolla, CA, USA) was used for performing the statistical analyses.

## Results

### Effects of co-administration of UDCA with RSV/EZE on liver fibrosis in the mouse model

**Figure 1.**
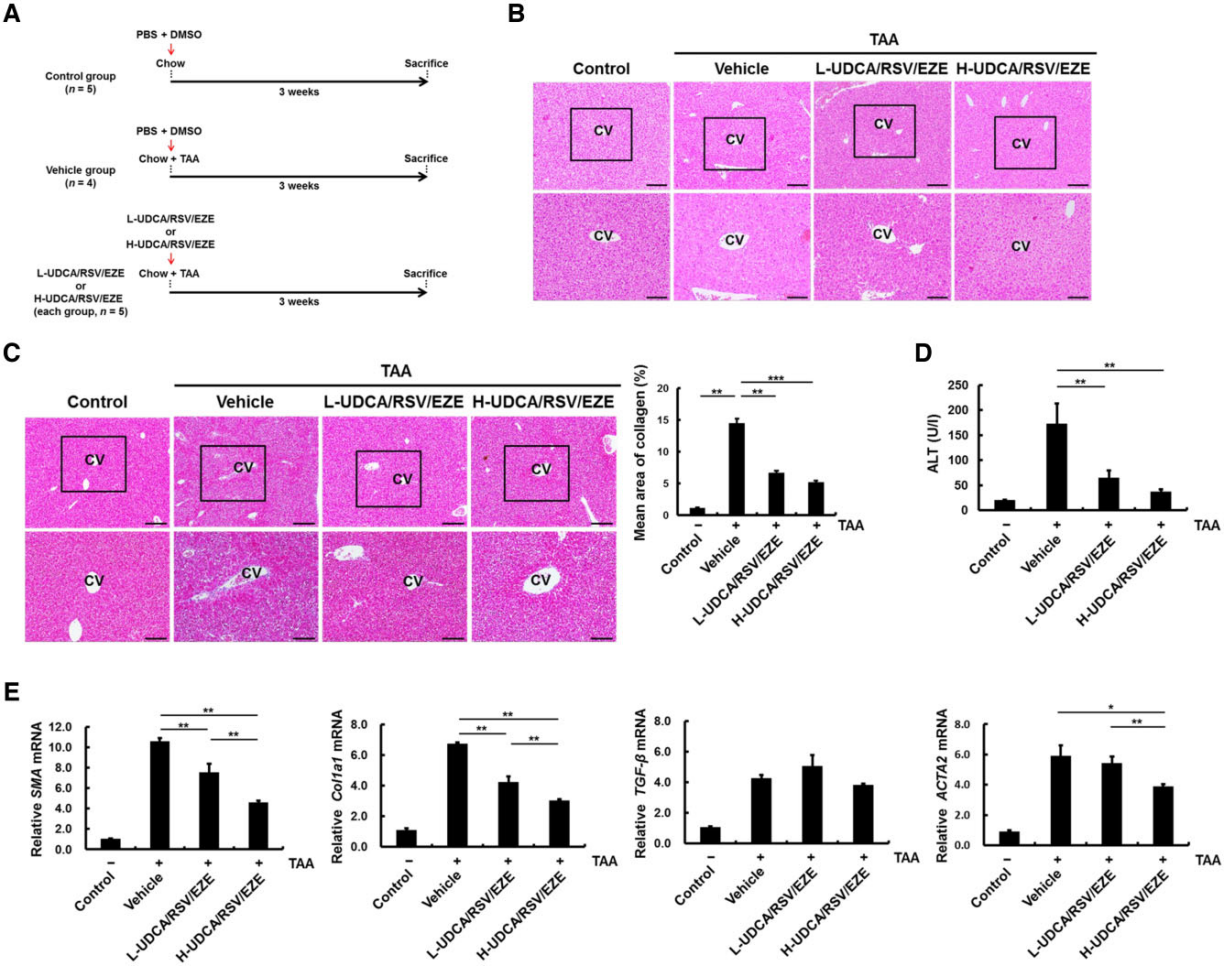
Antifibrotic effects of L-UDCA/RSV/EZE and H-UDCA/RSV/EZE in TAA-treated mice. (A) Mice were split into four groups: normal C57BL6/J mouse (control) (*n* = 5), vehicle (PBS/DMSO) (*n* = 4), and L-UDCA/RSV/EZE (*n* = 5), or H-UDCA/RSV/EZE (*n* = 5) groups. The treatment agents were orally administered 5 min prior to TAA administration (120 mg/kg, three times/week for 3 weeks) by intraperitoneal injection. (B) H&E and (C) Masson’s trichrome staining for histological examination on serial paraffin sections of each groups. Upper panel (scale bars = 200 µm) and lower panel (scale bars = 100 µm). (D) Effects of L-UDCA/RSV/EZE and H-UDCA/RSV/EZE on serum ALT levels. Levels of ALT were determined using chemical analyser. (E) mRNA levels of *SMA*, *Col1a1*, *TGF-β*, and *ACTA2* were determined by real-time PCR. Data are presented as the mean ± SD of at least three independent experiments. Statistical significance is indicated by *P < 0.05, **P < 0.01, ***P < 0.001. L-UDCA, low-dose ursodeoxycholic acid; H-UDCA, high-dose ursodeoxycholic acid; RSV/EZE, rosuvastatin/ezetimibe; TAA, thioacetamide; CV, central vein; ALT, alanine aminotransferase; PBS, phosphate buffered saline; DMSO, dimethyl sulfoxide; H&E, hematoxylin and eosin.

A schematic illustration of the treatment schedule employed for the development of TAA-treated mouse model is provided in Figure 1A. To minimize the stress caused by oral administration [30–32], PBS/DMSO, UDCA, and RSV/EZE were orally administered 5 min prior to TAA intraperitoneal injection thrice per week for 3 weeks. Following H&E staining, the number of ballooned hepatocytes in TAA-treated mice was slightly increased compared with that in the control mice, but those in the vehicle group, L-UDCA/RSV/EZE group, and H-UDCA/RSV/EZE groups showed no significant differences among each other (Figure 1B). In addition, collagen accumulation was increased in the vehicle group compared with that in the control group (*P* = 0.001), whereas L-UDCA/RSV/EZE (*P* = 0.001) and H-UDCA/RSV/EZE (*P* < 0.001) treatment significantly ameliorated collagen accumulation compared with that in the vehicle group (Figure 1C). The serum ALT levels of the L-UDCA/RSV/EZE (*P* = 0.006) and H-UDCA/RSV/EZE (*P* = 0.009) groups were significantly lower than those of the vehicle group in Figure 1D. The examination of the mRNA levels of *SMA*, collagen type 1 alpha 1 (*Col1a1*), transforming growth factor-beta (*TGF-β*), and actin alpha 2 (*ACTA2*) showed that TAA administration increased the mRNA levels in all tested groups compared with the levels in controls. However, H-UDCA/RSV/EZE treatment significantly decreased the mRNA levels of *SMA*, *Col1a1*, and *ACTA2* compared with the levels in the vehicle and L-UDCA/RSV/EZE groups (all *P* < 0.05), whereas mRNA levels of *TGF-β* were not significantly affected by L-UDCA/RSV/EZE and H-UDCA/RSV/EZE treatment (Figure 1E). These data suggest that L-UDCA/RSV/EZE and H-UDCA/RSV/EZE decreased collagen accumulation, serum ALT levels, and mRNA levels of fibrosis-related markers.

### Effects of co-administration of UDCA with RSV/EZE on the mouse liver subjected to acute lipogenic stimulation

**Figure 2.**
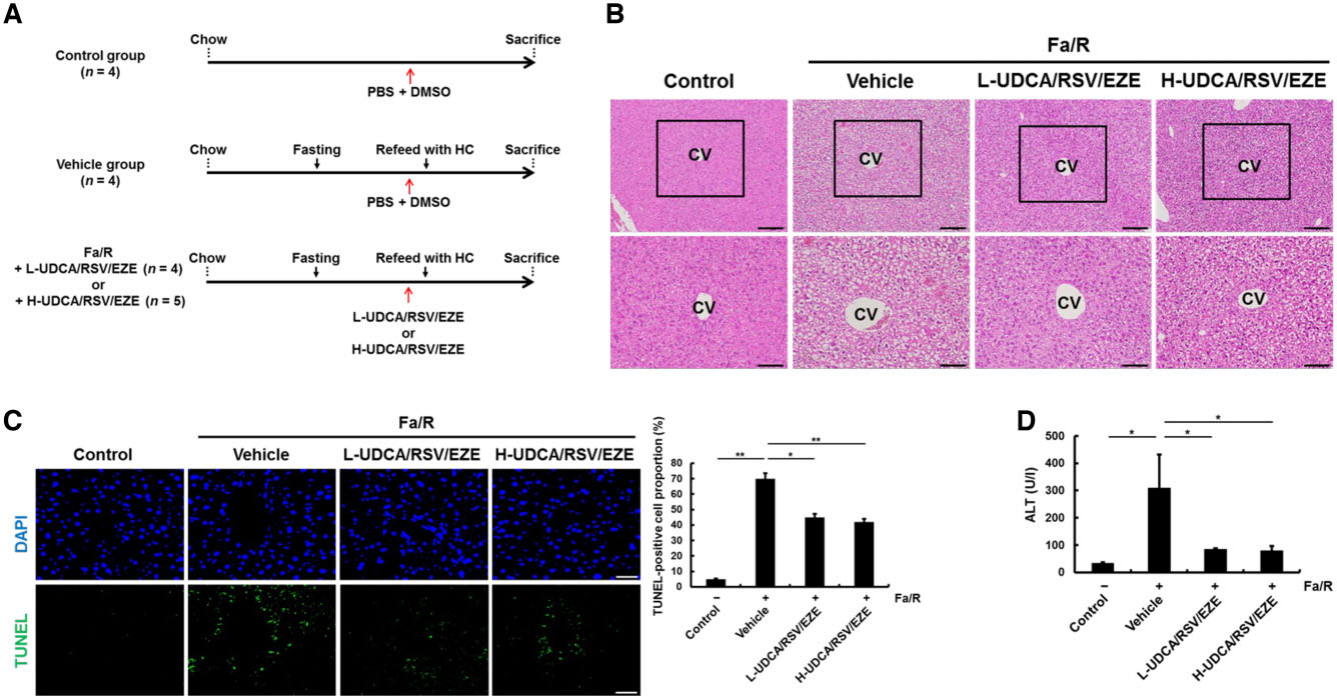
L-UDCA/RSV/EZE and H-UDCA/RSV/EZE protects the liver from acute lipogenic stimulation by refeeding after fasting in mice. (A) Schedule for the experiments on the fasting and refeeding mouse models. C57BL6/J male mice were divided into four groups: non-fasted mice treated with vehicle (PBS/DMSO) (control group) (*n* = 4), fasted mice followed by vehicle treatment prior to HCD refeeding (vehicle group) (*n* = 4), fasted mice followed by L-UDCA/RSV/EZE (*n* = 4), and H-UDCA/RSV/EZE (*n* = 5) treatment prior to HCD refeeding. After 18 h of refeeding, mice from all the groups were sacrificed. (B) Morphological analysis was performed on liver sections by H&E staining. Upper panel (scale bars = 200 µm) and lower panel (scale bars = 100 µm). (C) Apoptosis in liver sections from each group observed using TUNEL assay. Top panel: nuclei stained with 4',6-diamidino-2-phenylindole (DAPI) (scale bars = 100 µm), bottom panel: apoptotic cells stained with FITC (scale bars = 100 µm); right panel: quantitative analysis of TUNEL-positive cells. (D) Serum ALT levels in each group were determined using a chemical analyser. Data are presented as mean ± SD of at least three independent experiments. Statistical significance is indicated by *P < 0.05, **P < 0.01. Fa/R, fasting and refeeding; HCD, high-carbohydrate diet; L-UDCA, low-dose ursodeoxycholic acid; H-UDCA, high-dose ursodeoxycholic acid; RSV/EZE, rosuvastatin/ezetimibe; CV, central vein; ALT, alanine aminotransferase; PBS, phosphate buffered saline; DMSO, dimethyl sulfoxide; H&E, hematoxylin and eosin; TUNEL, terminal deoxynucleotidyl transferase dUTP nick end-labeling; FITC, fluorescein isothiocyanate.

A schematic illustration of the treatment schedule employed for the development of the fasting and HCD-refeeding mouse model is provided in Figure 2A. H&E staining revealed that the number of ballooned hepatocytes was increased by refeeding with a HCD, whereas L-UDCA/RSV/EZE and H-UDCA/RSV/EZE decreased the number of ballooned hepatocytes compared with that in the vehicle group (Figure 2B). In addition, the number of TUNEL-positive cells and serum ALT levels in the L-UDCA/RSV/EZE and H-UDCA/RSV/EZE groups were significantly decreased compared with those in the vehicle group (all *P* < 0.05) (Figure 2C and D). These data suggest that L-UDCA/RSV/EZE and H-UDCA/RSV/EZE treatment decreased liver damage of acute lipogenesis induced by fasting and HCD refeeding in a mouse model.

### Effects of co-administration of UDCA with RSV/EZE on the HFD-induced hepatic steatosis in a mouse model

**Figure 3.**
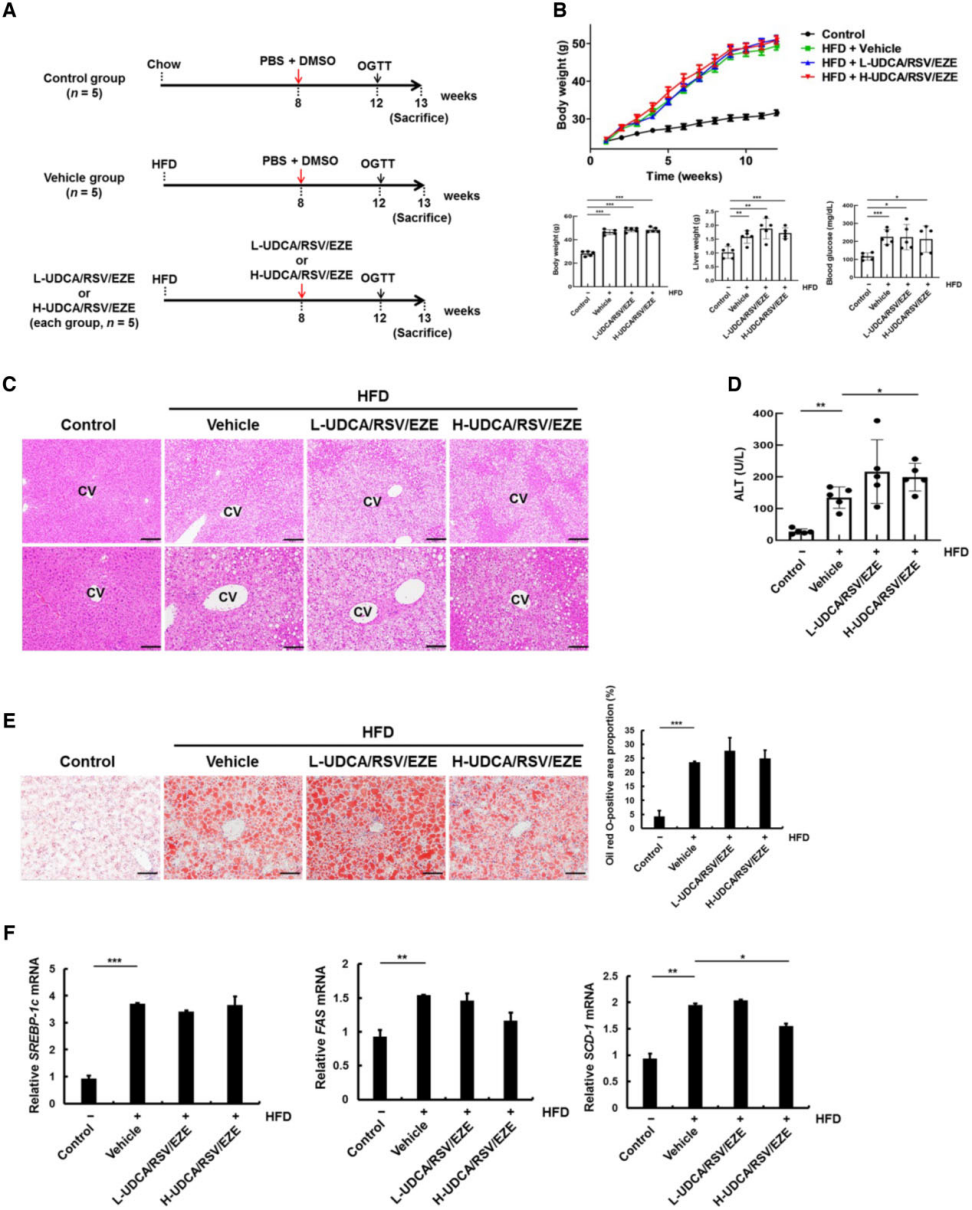
Effects of L-UDCA/RSV/EZE and H-UDCA/RSV/EZE on HFD-induced lipogenesis in a mouse model. (A) Schedule for the experiments on the HFD-fed mouse model. Mice were split into four groups: control (chow diet + PBS/DMSO), vehicle (HFD + PBS/DMSO), L-UDCA/RSV/EZE (HFD + L-UDCA/RSV/EZE), and H-UDCA/RSV/EZE (HFD + H-UDCA/RSV/EZE) groups (each group, *n* = 5). All treatments were performed three times/week for 5 weeks. (B) The line graph shows the body weight of mice for 12 weeks and bar graphs show the body weight, liver weight, and blood glucose levels of mice at the time of sacrifice. (C) Morphological analysis of the liver sections was carried out by H&E staining. Upper panel (scale bars = 200 µm) and lower panel (scale bars = 100 µm). (D) Serum ALT levels of each group were determined using a chemical analyser. (E) Lipid droplets in the tissue sections from each group were analysed by oil red O staining (scale bars = 100 µm). Right panel: quantitative analysis of oil red O-positive cells. (F) mRNA levels of lipogenesis-related proteins, namely *SREBP-1c*, *FAS*, and *SCD-1*, were determined using real-time PCR. Data are presented as the mean ± SD of at least three independent experiments. Statistical significance is indicated by *P < 0.05, **P < 0.01, ***P < 0.001. HFD, high-fat diet; L-UDCA, low-dose ursodeoxycholic acid; H-UDCA, high-dose ursodeoxycholic acid; RSV/EZE, rosuvastatin/ezetimibe; OGTT, oral glucose tolerance test; CV, central vein; ALT, alanine aminotransferase; PBS, phosphate buffered saline; DMSO, dimethyl sulfoxide; H&E, hematoxylin and eosin.

A schematic illustration of the treatment schedule employed for the development of the HFD-fed mouse model is given in Figure 3A. HFD increased the body weight (*P* < 0.001), liver weight (*P* = 0.002), and blood glucose level (*P* < 0.001) compared with the chow diet in control groups (Figure 3B). Histological analysis of H&E staining revealed that the number of aberrantly fatty hepatocytes was increased by HFD compared with the chow diet. Histological results showed no significant differences between the L-UDCA/RSV/EZE group and vehicle group. The number of ballooned hepatocytes decreased in the H-UDCA/RSV/EZE group compared with that in the vehicle group (Figure 3C). In addition, the serum ALT levels increased by HFD compared with those in the control group (*P* = 0.007), but UDCA/RSV/EZE treatment was not effective in reducing the serum ALT level (Figure 3D). Likewise, fat deposition (lipid droplets) increased in HFD groups compared with the control group (*P* < 0.001), but L-UDCA/RSV/EZE and H-UDCA/RSV/EZE had no prominent anti-obesity effects in the HFD-induced obese mice (Figure 3E). The mRNA expression of stearoyl-CoA desaturase 1 (*SCD-1*) decreased in the H-UDCA/RSV/EZE group compared with those in the vehicle group (*P* = 0.027), whereas the mRNA expression of *SREBP-1c* and *FAS* was not affected by H-UDCA/RSV/EZE treatment (Figure 3F). In addition, L-UDCA/RSV/EZE treatment had no prominent effects on the mRNA levels of *SREBP-1c*, *FAS*, and *SCD-1* (Figure 3F). These data showed that H-UDCA/RSV/EZE slightly decreased the number of ballooned hepatocytes and mRNA levels of *SCD-1*, but L-UDCA/RSV/EZE had no significant anti-obesity effects on the HFD-induced obesity in mice.

### Effects of co-administration of UDCA with RSV/EZE on the CDAHFD-induced NASH in a mouse model

**Figure 4.**
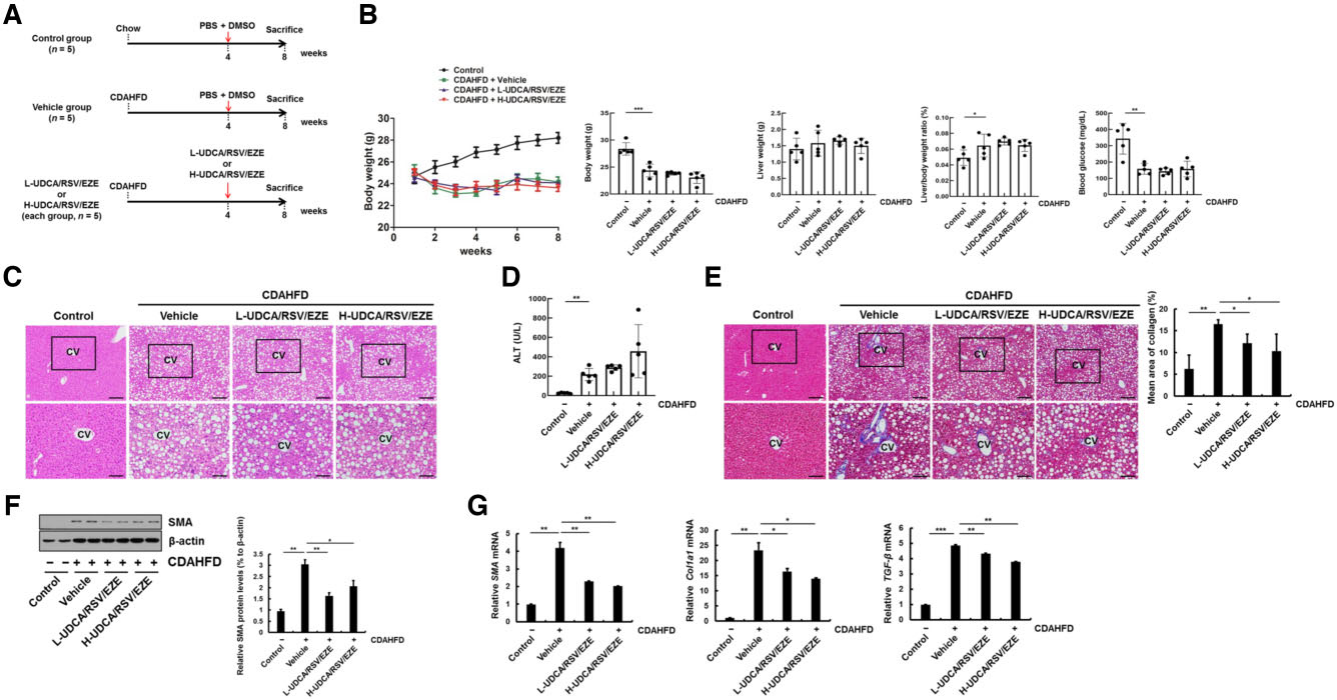
Effects of L-UDCA/RSV/EZE and H-UDCA/RSV/EZE on CDAHFD-induced NASH in a mouse model. (A) Schedule for the experiments on the CDAHFD-fed mouse model. Mice were split into four groups: control (chow diet + PBS/DMSO), vehicle (CDAHFD + PBS/DMSO), L-UDCA/RSV/EZE (CDAHFD + L-UDCA/RSV/EZE), and H-UDCA/RSV/EZE (CDAHFD + H-UDCA/RSV/EZE) groups (each group, *n* = 5). (B) Line graph showed the body weight of mice for 8 weeks and bar graphs showed the body weight, liver weight, and blood glucose of mice at time of sacrifice. (C) Pathological analysis of liver sections carried out by H&E staining. Upper panel (scale bars = 200 µm) and lower panel (scale bars = 100 µm). (D) Serum ALT levels of each group were determined using a chemical analyser. (E) Masson’s trichrome staining for histological examination on serial paraffin sections of each group. Upper panel (scale bars = 200 µm) and lower panel (scale bars = 100 µm). Right panel: quantitative analysis of collagen area. (F) Immunoblot assay of SMA for each group. Right: quantification results of immunoblot assay. (G) mRNA levels of *SMA*, *Col1a1*, and *TGF-β* were determined by real-time PCR. Data are presented as the mean ± SD at least three independent experiments. Statistical significance is indicated by *P < 0.05, **P < 0.01, ***P < 0.001. CDAHFD, choline-deficient L-amino acid-defined high-fat diet; L-UDCA, low-dose ursodeoxycholic acid; H-UDCA, high-dose ursodeoxycholic acid; RSV/EZE, rosuvastatin/ezetimibe; CV, central vein.

A schematic illustration of the treatment schedule employed for the development of the CDAHFD-fed mouse model is displayed in Figure 4A. CDAHFD decreased the body weight (*P* < 0.001) and blood glucose levels (*P* = 0.001), whereas the liver-to-body-weight ratio of the CDAHFD-fed mice was increased compared with that of the controls (*P* = 0.041) in Figure 4B. Histological analysis showed that mild fibrosis of the CDAHFD-fed mice was increased compared with that of the controls (Figure 4C) and serum ALT levels of CDAHFD-fed mice were increased compared with those of the controls (*P* = 0.001) in Figure 4D. In addition, collagen accumulation was increased in CDAHFD-fed mice compared with that in the controls (*P* = 0.002), but collagen accumulation significantly decreased in L-UDCA/RSV/EZE (*P* = 0.015) and H-UDCA/RSV/EZE (*P* = 0.027) groups compared with that in the vehicle group in Figure 4E. Moreover, CDAHFD increased the protein level of SMA compared with the levels in the controls (*P* = 0.002), but L-UDCA/RSV/EZE (*P* = 0.007) and H-UDCA/RSV/EZE (*P* = 0.026) significantly decreased the protein level of SMA compared with the observation in the vehicle group (Figure 4F). In addition, mRNA levels of *SMA*, *Col1a1*, and *TGF-β* were increased in CDAHFD-fed mice compared with the controls (all *P* < 0.01), whereas L-UDCA/RSV/EZE and H-UDCA/RSV/EZE significantly decreased the mRNA levels of *SMA* (all *P* < 0.01), *Col1a1* (all *P* < 0.05), and *TGF-β* (all *P* < 0.01) compared with those in the vehicle group (Figure 4G). Overall, L-UDCA/RSV/EZE and H-UDCA/RSV/EZE treatment decreased liver fibrosis in a NASH mouse model induced by CDAHFD.

### UDCA augmented anti-lipotoxic effects of RSV/EZE in an *in vitro* model of NAFLD

**Figure 5.**
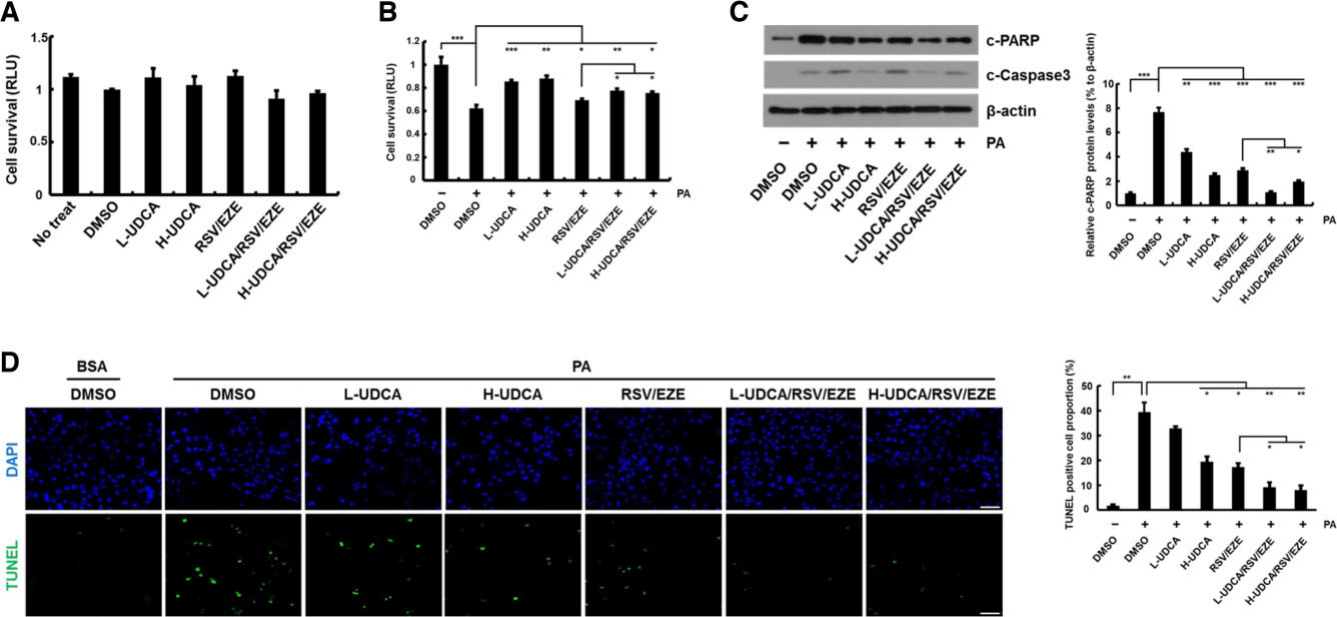
Anti-lipotoxic effects of UDCA combined with RSV/EZE in an *in vitro* model of NAFLD. (A) The cytotoxic effect of L-UDCA (100 μM), H-UDCA (200 μM), RSV/EZE (25 μM), and their combination observed on Hepa1c1c7 cells for 18 h. (B) Cell survival was determined via cell viability assay using a Cell Titer-Glo assay kit. After treatment with PA (500 μM) for 18 h, Hepa1c1c7 cells were treated with L-UDCA, H-UDCA, RSV/EZE, and their combination for 18 h. (C) Western blot analysis of aggregated proteins of Hepa1c1c7 cells. Hepa1c1c7 cells were treated as shown in Figure 5B. (D) Apoptotic cells were observed using the TUNEL assay (scale bars = 100 μm). Hepa1c1c7 cells were treated as shown in Figure 5B. Statistical significance is indicated by *P < 0.05, **P < 0.01, ***P < 0.001. L-UDCA, low-dose ursodeoxycholic acid; H-UDCA, high-dose ursodeoxycholic acid; RSV/EZE, rosuvastatin/ezetimibe; PA, palmitic acid; RLU, relative light unit; DMSO, dimethyl sulfoxide; BSA, bovine serum albumin; TUNEL, terminal deoxynucleotidyl transferase dUTP nick end-labeling.

To identify the effects of UDCA on the anti-lipotoxicity of RSV/EZE, the effects of L-UDCA (100 μM) or H-UDCA (200 μM) combined with RSV/EZE (25 μM) were examined. The cell numbers were not significantly different among groups treated with DMSO, UDCA, and RSV/EZE (Figure 5A). Treatment with PA resulted in the loss of viability of Hepa1c1c7 cells (*P* < 0.001). However, UDCA, RSV/EZE, and their combination significantly restored Hepa1c1c7 cell viability compared with PA treatment (all *P* < 0.05) in Figure 5B. In addition, UDCA, RSV/EZE, and their combination significantly decreased the expression of c-PARP (all *P* < 0.01) and c-Caspase 3 compared with that under PA treatment. Particularly, the expression of c-PARP (all *P* < 0.05) and c-Caspase 3 was lower in cells treated with UDCA in combination with RSV/EZE than that in cells treated with RSV/EZE alone in Figure 5C. The number of TUNEL-positive cells in the H-UDCA, RSV/EZE, L-UDCA/RSV/EZE, and H-UDCA/RSV/EZE treatment groups was significantly decreased compared with that in the PA-treated-with-DMSO group (all *P* < 0.05) in Figure 5D. Moreover, UDCA/RSV/EZE combinations treatment synergistically reduced apoptotic cells compared with RSV/EZE treatment alone (all *P* < 0.05). These findings suggest that UDCA effectively augmented the anti-lipotoxic effects of RSV/EZE in the *in vitro* model of NAFLD when compared with RSV/EZE treatment alone.

## Discussion

To date, no effective therapeutic agents have been approved for NAFLD [33]. Although UDCA has been used to treat cholestatic liver disease, primary biliary cirrhosis, and primary sclerosing cholangitis, UDCA monotherapy often shows a suboptimal response, indicating the need for additional therapies [34, 35]. In addition, Meeberg *et al.* [36] showed that H-UDCA mediates more potent improvements in liver functions than L-UDCA in patients with cystic fibrosis with cholestatic liver disease. However, Smith *et al.* [37] determined that L-UDCA is associated with prominent biochemical improvements, while H-UDCA is associated with an approximately 2-fold increase in serious events. Although UDCA has been demonstrated as a useful agent, no consensus has been established with respect to the drug dosage and its effect. Statin/EZE therapy can be considered effective for hypercholesterolemia and liver diseases [38, 39]. However, the relationship between statin/EZE and NAFLD remains poorly understood. Accordingly, we evaluated the potential therapeutic effect of the combined use of L- or H-UDCA and a fixed dose of RSV/EZE in several NAFLD mouse models. Accordingly, the present study showed hepatoprotective effects of L- and H-UDCA in conjunction with RSV/EZE.

In our study, we first found that H-UDCA/RSV/EZE treatment decreased serum ALT levels and mRNA levels of fibrosis-related factors compared to L-UDCA/RSV/EZE treatment in TAA-induced liver fibrosis. Second, L-UDCA/RSV/EZE and H-UDCA/RSV/EZE treatments decreased serum ALT levels and apoptosis during acute lipogenesis induced by fasting and refeeding with an HCD. Third, H-UDCA/RSV/EZE treatment was more effective in reducing hepatocyte ballooning and mRNA expression of *SCD-1* compared with L-UDCA/RSV/EZE treatment in HFD-induced hepatic steatosis. Fourth, L-UDCA/RSV/EZE and H-UDCA/RSV/EZE treatments decreased collagen accumulation and fibrosis-related markers in CDAHFD-induced fibrosis. Fifth, UDCA combined with RSV/EZE restored cell viability and decreased apoptosis rates, thereby acting against lipotoxicity-mediated by PA in Hepa1c1c7 cells compared with RSV/EZE treatment alone.

Our study had several unique findings. First, our study confirmed the hepatoprotective effects of L-UDCA/RSV/EZE and H-UDCA/RSV/EZE in TAA-induced liver injury. Previous studies have shown that TAA administration is a commonly used toxic agent to induce NASH in rodents [40–42]. Likewise, we showed TAA-induced liver injury through histological, blood biochemical analysis and fibrosis-related markers in mice. In our study, L-UDCA/RSV/EZE and H-UDCA/RSV/EZE had no significant antifibrotic effects in H&E staining, but L-UDCA/RSV/EZE and H-UDCA/RSV/EZE treatment significantly decreased collagen accumulation in Masson’s trichrome staining. In addition, H-UDCA/RSV/EZE significantly ameliorated ALT levels and fibrosis-related mRNA expression against TAA-induced hepatotoxicity compared with the vehicle and L-UDCA/RSV/EZE groups. Rolandi *et al.* [43] showed that UDCA significantly decreased serum ALT levels in patients with chronic active hepatitis and Kong *et al.* [44] confirmed that nutria bile had a hepatoprotective effect in a TAA-induced liver injury in mice. Based on these results, we found that L-UDCA/RSV/EZE and H-UDCA/RSV/EZE treatment improved hepatic function and reduced fibrosis-related marker expression, and H-UDCA/RSV/EZE might have a better hepatoprotective effect than L-UDCA/RSV/EZE against TAA-induced liver injury in mice.

Second, we found that L-UDCA/RSV/EZE and H-UDCA/RSV/EZE treatment mitigated fasting and HCD-refeeding-induced liver damage by decreasing ballooning degeneration, apoptotic cell death, and serum ALT levels. Previous studies found that fasting and refeeding with a HCD increased fatty acid synthesis, followed by hepatocyte apoptosis and hepatocyte ballooning [29, 45–47]. In addition, Zhang *et al*. [48] found that increased serum free fatty acid levels were positively correlated with serum ALT levels, and Rodrigues *et al.* [49] and Amaral *et al.* [50] showed that UDCA prevents apoptotic cell death associated with cholestasis. Likewise, our study revealed that ballooned hepatocytes, apoptotic cells, and serum ALT levels were increased by refeeding with an HCD after fasting but significantly recovered in the L-UDCA/RSV/EZE and H-UDCA/RSV/EZE treatments. Based on these data, we suggest that L-UDCA/RSV/EZE and H-UDCA/RSV/EZE treatment can protect against fasting and HCD-refeeding-induced liver injury.

Third, we showed that H-UDCA/RSV/EZE treatment attenuated hepatocellular ballooning and lipogenesis-related markers in a mouse model of HFD-induced obesity. However, we found that serum ALT levels and lipid droplets were slightly increased in the L-UDCA/RSV/EZE- and H-UDCA/RSV/EZE-treated groups. In previous studies, Zhang *et al.* [51] found that UDCA administration ameliorates metabolic dysfunction in mice with HFD-induced mice, and Pathil *et al.* [52] determined that UDCA-conjugated phospholipids alleviate serum ALT levels in HFD- and MCD-induced liver injury in mice. In our data, although serum ALT levels were increased in the L-UDCA/RSV/EZE and H-UDCA/RSV/EZE groups, this ALT increase was relatively small, with a wide variation in ALT levels in the L-UDCA/RSV/EZE-treated group. Similar findings were observed in a study by Saeedi *et al.* [53], who found that ALT levels were slightly increased in the RSV/EZE-treated group, but this increase was relatively small and not likely to be clinically relevant. Consequently, we showed that H-UDCA/RSV/EZE treatment increased liver function by attenuating ballooned hepatocytes and fibrosis-related markers, but the anti-obesity effects of the UDCA–RSV/EZE combination should be further studied in HFD-induced obesity.

Fourth, we showed that L-UDCA/RSV/EZE and H-UDCA/RSV/EZE treatment increased liver function in CDAHFD-induced liver fibrosis. The CDAHFD mouse model is regarded as a mouse model of NASH-associated liver fibrosis [54, 55]. In a previous study, Suga *et al.* [55] found that total bile acid concentration in the bile was significantly decreased at 9 weeks of CDAHFD feeding and increased in the peripheral plasma for 3–15 weeks of CDAHFD feeding, suggesting that the change in bile-acid-metabolizing enzymes may promote liver fibrosis. In our study, we determined that feeding CDAHFD increased liver damage, but L-UDCA/RSV/EZE and H-UDCA/RSV/EZE treatment attenuated collagen accumulation and fibrosis-related protein and mRNA levels. Although the serum ALT levels of the H-UDCA/RSV/EZE-treated group seemed to increase, their variation in the H-UDCA/RSV/EZE group was large without statistical significance. Collectively, our results revealed that L-UDCA/RSV/EZE and H-UDCA/RSV/EZE treatment protected the liver against CDAHFD-induced NASH.

Fifth, UDCA significantly augmented the anti-lipotoxic effect of RSV/EZE in the *in vitro* model of NAFLD. PA plays crucial roles in the pathogenesis of NAFLD [56]. The mouse hepatoma cell line (Hepa1c1c7 cells) is a suitable *in vitro* model for the examination of liver diseases owing to the efficient recapitulation of liver phenotype under cultured conditions [57]. To assess the anti-lipotoxic effects of UDCA, we employed an *in vitro* model of NAFLD developed using PA-treated Hepa1c1c7 cells. Feldstein *et al.* [58] found that caspase 3 activation and hepatocyte apoptosis are major features of NAFLD. In addition, Witek *et al.* [59] showed that pharmacological pan-caspase inhibitor VX-166 may reduce the development of fibrosis in the NASH mouse model by inhibition of hepatic apoptosis. Derdak *et al.* [60] determined that pifithrin-α-p-nitro, a p53 inhibitor, reduced steatosis and apoptosis in mouse models of NAFLD. Likewise, our results showed that UDCA combined with RSV/EZE significantly restored cell viability and reduced the levels of apoptosis-related markers compared to RSV/EZE treatment alone in an *in vitro* model of NAFLD.

Despite the strengths of our study, we are also aware of the issues that remain unresolved. First, because our study results are based on an *in vivo* model, our results should be further validated in human studies. However, although the mechanism should be further investigated in the NAFLD model, our results with diverse mouse models might indicate that UDCA and RSV/EZE combination treatment ameliorates liver fibrosis. Second, we found unexpected findings such as ALT elevation and an increase in lipid droplets in some models, which should be further investigated to explain these findings. Third, although we found that UDCA combined with RSV/EZE showed favorable influences in diverse models of NAFLD, the underlying mechanism for this finding should be further investigated.

In conclusion, our study showed that the hepatoprotective effects of UDCA treated with RSV/EZE may be a novel therapy to better protect NAFLD progression.

## Authors’ Contributions

S.H.B. and S.U.K. conceived and designed the project. S.H.S. and D.H.L. analysed the data. S.U.K., S.H.S., and D.H.L. wrote the manuscript. Y.S.L., K.J.C., H.J.P., H.W.L., B.K.K., J.Y.P., D.Y.K., and S.H.A. provided critical insights into manuscript preparation. All authors read and approved the final manuscript.

## Funding

This study was supported by Daewoong Pharmaceutical company, and was supported by a Faculty Research Grant from the Yonsei University College of Medicine (6-2019-0068 to S.H. Bae), by a grant from the Korea Health Technology R&D Project through the Korea Health Industry Development Institute (KHIDI), funded by the Ministry of Health & Welfare, Republic of Korea (HI17C0913 and HI16C0257 to S.H. Bae). In addition, this work was supported by the National Research Foundation of Korea (NRF) grant funded by the Korea government (MSIT) (NRF-2022R1A2C2003438 to S.H. Bae; NRF-2021R1C1C2095694 to D.H. Lee), and by Basic Science Research Program through the National Research Foundation of Korea (NRF) funded by the Ministry of Science, ICT & Future Planning (2019R1A2C4070136 to S.U. Kim). The funders had no role in study design, data collection and analysis, decision to publish, or preparation of the manuscript.

## Conflict of Interest

The authors declare that there is no conflict of interests in this study.
